# Infections in Burn Patients: A Retrospective View over Seven Years

**DOI:** 10.3390/medicina58081066

**Published:** 2022-08-08

**Authors:** Savas Tsolakidis, David Lysander Freytag, Elisabeth Dovern, Ziyad Alharbi, Bong-Sung Kim, Khosrow Siamak Houschyar, Georg Reumuth, Benedikt Schäfer, Hans-Oliver Rennekampff, Norbert Pallua, Gerrit Grieb

**Affiliations:** 1 Department of Plastic Surgery and Hand Surgery, Burn Center, Medical Faculty, RWTH Aachen University Hospital, Pauwelsstrasse 30, 52074 Aachen, Germany; 2 Austrian Cluster of Tissue Regeneration, Ludwig Boltzmann Institute for Experimental and Clinical Traumatology at the Research Centre for Traumatology of the Austrian Workers Compensation Board (AUVA), Donaueschingenstraße 13, 1200 Vienna, Austria; 3 Millesi Center for Surgery of Peripheral Nerves, Vienna Private Clinic, Pelikangasse 15, 1090 Vienna, Austria; 4 Department of Plastic Surgery and Hand Surgery, Gemeinschaftskrankenhaus Havelhoehe, Kladower Damm 221, 14089 Berlin, Germany; 5 Plastic Surgery and Burn Unit, Fakeeh Care Hospitals & Fakeeh College for Medical Sciences, Jeddah 21461, Saudi Arabia; 6 Department of Plastic Surgery and Hand Surgery, University Hospital Zurich, 8091 Zurich, Switzerland; 7 Department of Dermatology and Allergology, Medical Faculty, RWTH Aachen University Hospital, Pauwelsstrasse 30, 52074 Aachen, Germany; 8 Department of Plastic Surgery and Hand Surgery, Evangelische Elisabeth Klinik, Luetzowstrasse 26, 10785 Berlin, Germany; 9 Department of Plastic, Aesthetic and Burn Surgery, Rhein-Maas Klinikum, Mauerfeldchen 25, 52146 Würselen, Germany

**Keywords:** burn, infection, antibiotics

## Abstract

*Backgroundand objectives:* Burn patients represent a challenging cohort because the injuries entail a vulnerability to colonisation by microorganisms. The ensuing infections can lead to serious complications and, in many cases, to the death of the burn patient. Surgical intervention and wound dressings, as well as antibiotic treatment, are crucial for optimising the treatment of the patient. *Material* *and Methods:* In this retrospective analysis, we analysed the treatment course, antibiotic therapy, and general complications of 252 burn patients with second- or third-degree burns over a time span of 7 years. *Results:* Patients who developed infections tended to have, on average, a higher total body surface area (TBSA), higher abbreviated burn severity index (ABSI) scores, and longer hospital stays. Patients who were admitted to the burn unit after 2006 had significantly shorter stays in the burn unit. TBSA and ABSI scores were lower in the patient cohort admitted after 2006. Patients exhibiting a TBSA greater than 30% had significantly longer hospital stays and antibiotic treatment periods. TBSA and ABSI scores were significantly higher in patients who died. The results of binary logistic regression indicate that a higher ABSI score increases the odds ratio of developing an infection. Bacteria number had no significant effect on the odds of patient death but positively influenced the odds ratio of developing an infection. TBSA was negatively associated with the risk of developing an infection and was an insignificant predictor of mortality. *Conclusions:* To gauge the optimal treatment for a burn patient, it is crucial for practitioners to correctly select, dose, and time antibiotics for the patient. Monitoring bacterial colonisation is vital to nip rising infection in the bud and ensure the correct antibiotic selection. This will help prevent the development of multi-resistant bacteria.

## 1. Introduction

Dealing with severely burned patients in a burn unit is challenging. Not only should adequate emergency treatment be achieved in the first 24 h, but well-considered infection control precautions should also be carried out [1].

Physiologically, burns represent a state of emergency for the main functions of the body with potential life-threatening side effects. Hypermetabolism, bacterial inoculation, and a complete breakdown of the natural barrier are the first problem areas to arise. Satisfactory treatment may not be achieved without profound expertise and experience in dealing with burn patients. Treating severely burned patients entails the treatment of different potential infections. On the one hand, antibiotic treatment during infection is mandatory and, at times, lifesaving. On the other hand, inappropriate antibiotic administration can foster resistance to antibiotics, accelerate the development of multi-resistant germs, and negatively impact the ecosystem of the intensive care unit.

As the pharmacokinetics and pharmacodynamics in burn patients are altered by the specific pathophysiology of burn trauma, the correct and adequate dosage of an antibiotic scheme varies on a case-by-case basis.

Infections in burn patients can occur due to a plethora of reasons. Initial colonisation of the affected burn areas by Gram-positive and Gram-negative bacteria from different origins in the first seven days after injury plays a key role in the development of further infections [2]. The transition from colonisation to infection depends mainly on three factors: the extent of bacterial colonisation; the immuno-defence of the patient; and the virulence of the pathogen [2].

Delayed operative treatment and wound coverage can promote conversion from early colonisation to invasive systemic infection, namely, burn wound sepsis, of the affected areas with nosocomial pathogenic agents. This, in turn, can provoke further complications [3]. Topical anti-infective therapy with an agent able to penetrate the eschar and early necrotomy is critical.

Standard diagnoses for infection and sepsis do not apply to burn patients. Severe burns can lead to various inflammatory response pathways, which complicates the diagnosis of an acute infection [4]. Some authors have attempted to establish specific antibiotic therapy guidelines based on infection and sepsis definitions set by the American Burn Association in 2007 [5]. Considering the diversity of the microbiological ecosystem in burn centres worldwide and individual patient differences, general recommendations or even therapeutic formulas for antibiotics are challenging or even hazardous.

In this retrospective study, we highlight our treatment course, treatment outcomes, and complications in 252 patients who were admitted to the burn unit of the RWTH Aachen University Hospital over a time span of 7 years. With these results, we hope to contribute to a better understanding of how treatment in a burn unit works, what kind of complications a burn unit may see on a day-to-day basis, and how our treatment algorithms can be optimised.

## 2. Materials and Methods

### 2.1. Subjects

This retrospective study included 252 burn patients with second- and third-degree burns admitted to the burn unit of the RWTH Aachen University Hospital between 2002 and 2009. The age of the patients ranged from 19 to 97 years, with a median age of 39 years (28–56), and the majority of the patients were male (*n* = 176 [69.8%]). Patients with haematological diseases, cancer, or Acquired Immunodeficiency Syndrome (AIDS) were excluded from the study. On admission, the proportion of burnt total body surface area (TBSA) of each patient was measured, as well as the patient’s Abbreviated Burn Severity Index (ABSI) score [6]. This score represents a prognostic value of the probability of survival after a burn injury and includes the parameters: gender, age, the proportion of burnt body surface area, third-degree burn, and inhalation injury. A score of 2–3 points implies a chance of survival of 99%, while a score of 13 represents a chance of survival below 1%.

Inhalation injury was diagnosed on admission by fibreoptic bronchoscopy. Patients with inhalation injury, COHb levels > 10%, and/or neurological deficits were treated with hyperbaric oxygen therapy (HBO Zentrum Euregio Aachen GmbH & Co. KG, Aachen, Germany).

Daily disinfection of the affected body areas and sterile dressing changes with gauze and antiseptic solution (polyhexanide and mafenidacetate) were performed.

To track bacterial colonisation in the patients, chest X-rays and standardised blood tests measuring leucocyte number, C-reactive protein (CRP), and procalcitonin (PCT) were performed daily [1]. A leucocyte count of >10.0/nL, a CRP serum concentration of >5 mg/L, and a procalcitonin concentration of >0.5 ng/mL were considered elevated. Out of all the above-mentioned laboratory markers, procalcitonin is the most reliable indicator of infection due to its specificity and early increase in imminent infections [7].

Microbial swabs of the affected burn areas were taken on admission, as well as in the following days before disinfection and daily dressing changes. Furthermore, nasal, inguinal, oral, and perianal swabs were obtained on admission, as well as biweekly, to identify patients with multi-resistant bacteria. Blood cultures and samples of the tracheal exudate were taken if sepsis was suspected.

In cases where surgical intervention was necessary, the first surgical treatment performed on the patients was the removal of eschar. Depending on depth, patient status, and the extent of injury, both tangential excision of the burned tissue and early wound closure by skin grafts were performed. In patients with deep circular second- and third-degree burns, escharotomies or even fasciotomies were performed on admission. In patients with second-degree burns, additional biosynthetic wound dressings, such as Suprathel (Polymedics, Denkendorf, Germany) or Biobrane (Smith&Nephew, Tuttlingen, Germany), were used to avoid daily dressing changes. All surgical procedures on admission were carried out in the isolated and heatable operating theatre in our burn unit.

Infection was diagnosed in the early phase through reliable clinical criteria. These were: specific changes in wound secretion, pus in the wounds, odours, necrosis, colour changes, or a minimum of 3 of the infection parameters defined by the ABA (Table 1) [5]. In the case of no clinical infection, perioperative antibiotics were administered to the patient as single-shot infection prophylaxis during the initial surgical procedure.

In addition, a documented infection is characterised by one or more of the following:

1. Culture-positive infection;
2. Pathologic tissue source identified;
3. Clinical response to antibiotic treatment.

In cases of wound infection, an early broad-spectrum antibiotic was given intravenously through a central venous catheter by continuous infusion. After receiving positive swab results from burn wounds, the antibiotic was adjusted according to the antibiogram. Combinations of antibiotics were only administered to fight multi-resistant strains according to the antibiogram. For patients with diagnosed pneumonia, antibiotic treatment was administered for seven days, while the clinical situation was re-evaluated on the third day after the first application. In bloodstream sepsis and bloodstream fungal infections, the antibiotic agent was applied for 10 additional days following the first negative microbial result.

If comorbidity was identified in a patient on admission, the antibiotic treatment was selected on an empirical basis. For example, we administered piperacillin/tazobactam as a first-line empiric antibiotic when there was a clinical suspicion of early systemic infection but microbiology results had not yet been obtained or a causative agent had not been isolated.

Patients with multi-resistant organisms were isolated, and healthcare workers had to wear gowns, masks, and gloves according to the hygienic protocol of the RWTH Aachen University Hospital. Daily assessment of the wounds at each dressing change was performed according to a standard protocol.

Intravascular devices were changed when signs of local infection arose or when the systemic infection focus could not be localized and when central catheters were older than five days.

### 2.2. Analysis Strategy and Statistics

To gain greater insight into the type of patient admitted to the burn unit of the RWTH Aachen University Hospital in the period of study, descriptive statistical analysis was carried out first. Whitney–Mann U tests (Wilcoxon Rank-Sum) were then carried out to find statistically significant differences between patients who developed an infection and those who did not, those who had a TBSA greater than 30% and those with a TBSA smaller than 30%, those who were admitted before 2005 and those admitted after, and lastly, patients who died and those who did not. Simple checks revealed that the outcomes for many variables within our dataset were very skewed. For example, the hospital stay duration was skewed towards the shorter end, with most patients spending less than 36 days in the burn unit.

Thereafter, to examine whether there were any notable correlations within the patient cohort, linear regression was carried out on the following parameters: stay duration and bacteria number, TBSA, ABSI, and inhalation injury; antibiotic treatment duration and bacteria number and pulmonary infiltrate. To ensure our results from the linear regression analyses were as clear as possible, assumption tests were carried out to ensure the independence of variables, the absence of collinearity and influential outliers, and the independence and constant variance of residuals. Both regression models fulfilled the assumptions satisfactorily—albeit not perfectly. This is specified later in the Results section.

Lastly, to examine how ABSI, TBSA, and the bacteria number affect the odds of developing an infection, as well as the odds of dying, from the data at hand, we carried out binary logistic regression. The largest challenge to the validity of these results is the relatively small number of observations. Conventionally, an *n* of at least 500 is recommended for binary logistic regression. Nonetheless, Field and Gillett [8] and Leblanc and Fitzgerald [9] argue that fewer observations (50 and 30, respectively) are needed per independent variable for results to be satisfactory.

## 3. Results

### 3.1. General Subject Cohort

In this retrospective study, we studied 252 patients with moderate to severe second- to third-degree burns. The median total burned body surface area (TBSA) of the patients was 15% (6–24). The median Abbreviated Burn Severity Index (ABSI) score was 5 (4–7), which predicts a chance of survival of 90–99%.

**Figure 1:**
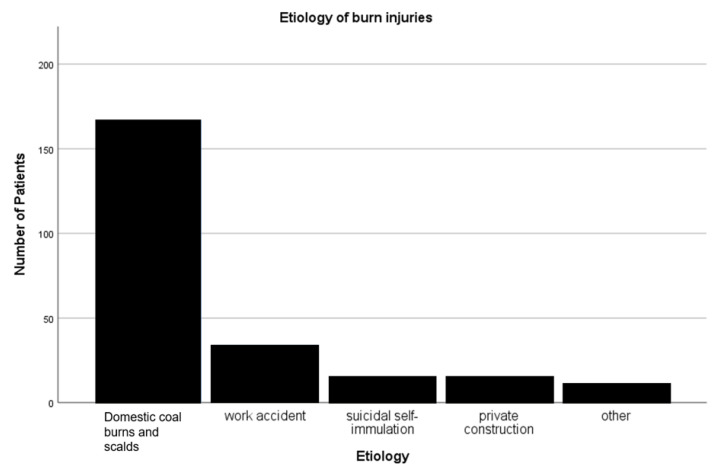
Aetiology of burn injuries.

The leading cause of burn injury in the patient cohort was domestic coal burns and scalds, followed by work accidents and suicidal self-immolation (Figure 1). Eighty-one patients (32.1%) had to be intubated on the basis of an inhalation injury on admission and/or the development of pneumonia during their hospital stay. These patients had a median intubation interval of 9 days (1.25–22.75). The median duration of stay in the burn unit of all included patients was 18 days (8–33.75).

**Figure 2:**
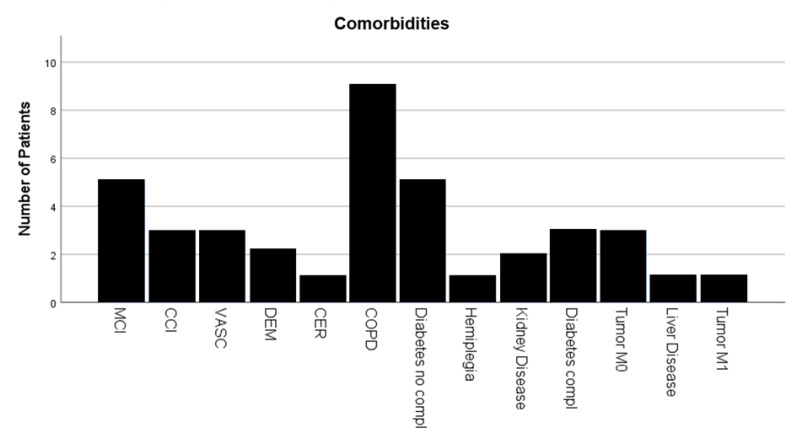
Number of Comorbidities. MCI = myocardial infarction; CCI = congestive cardiac insufficiency; VASC = peripheral vascular disease; DEM = dementia; CER = cerebrovascular disease; COPD = chronic obstructive pulmonary disease; Tumour M1 = malignant tumour with metastasis.

The majority of the patients (84.5%) had no comorbidities recorded on admission. About 10 patients (4%) had chronic obstructive lung disease and asthma in their medical history (Figure 2).

Fifty-four patients developed general complications other than infection during their hospital admittance. Most commonly, these complications were depressive episodes, followed by non-ST-elevation myocardial infarction.

Over the 7-year time span, the most commonly found bacteria were coagulase-negative Staphylococcus (12.2%, Gram-positive), followed by *Pseudomonas aeruginosa* (Gram-negative) and *Staphylococcus aureus* (Gram-positive) (both 10.6%), *Enterococcus* (9.4%, Gram-positive), and *E. coli* (8.6%, Gram-negative).

Not all wounds with a proven presence of bacteria resulted in an active infection. Out of all of the first microbiological tests performed on the 252 patients at admission, 89 came back positive (35.3%). Within this group, the predominantly found bacteria were *Staphylococcus aureus* (14.6%, Gram-positive), coagulase-negative Staphylococcus (13.5%, Gram-positive), *Pseudomonas aeruginosa* (10.1%, Gram-negative), and *Enterococcus* (9%, Gram-positive). Of the 89 patients, 78 did not develop any form of wound infection. Additionally, this group did not develop a wound infection despite the, on average, three (1.5–4) different types of bacteria present in the burn wound. The most commonly found bacteria in the burn wounds that did not develop a wound infection were coagulase-negative Staphylococcus (12.7%, Gram-positive), *Staphylococcus aureus* (10.9%, Gram-positive), *Pseudomonas aeruginosa* (10.5%; Gram-negative), *Enterococcus* (9.6%, Gram-positive), and *E. coli* (8.6% Gram-negative)

Table 2 demonstrates the ranking of the most common microbial strains in burn wounds.

### 3.2. The Infected Burn Patient

A total of 195 patients (77.4%) showed no signs of infection during their treatment course. The median ABSI score of this patient group was 5 (4–7), corresponding to a chance of survival of 90–99%. The median TBSA was 11.25% (5–20.25), while the median duration of stay in the burn unit was 13 days (6–26). Fifty-seven patients (22.6%) had at least one diagnosed and treated focus of infection. In these patients, the median ABSI score on admission was 7 (6–8), corresponding to a chance of survival of 80–90%; the median total burned surface area (%TBSA) was 21.5% (15–28.5), and the median duration of treatment in the burn unit was 38 days (25.5–60.5). The Whitney–Mann U Test run to examine whether these variations between the infected and non-infected groups differed significantly returned a positive result. Both the ABSI score (*p* < 0.001) and TBSA (*p* < 0.001) were significantly higher in patients who developed an infection (ABSI mean rank = 172.89; TBSA mean rank = 163.70) than in those who did not (ABSI mean rank = 112.22; TBSA mean rank = 114.92). The hospital stay for those who developed an infection was also significantly higher (mean rank = 192.89) than the stay for patients who did not (mean rank = 197.09).

**Figure 3:**
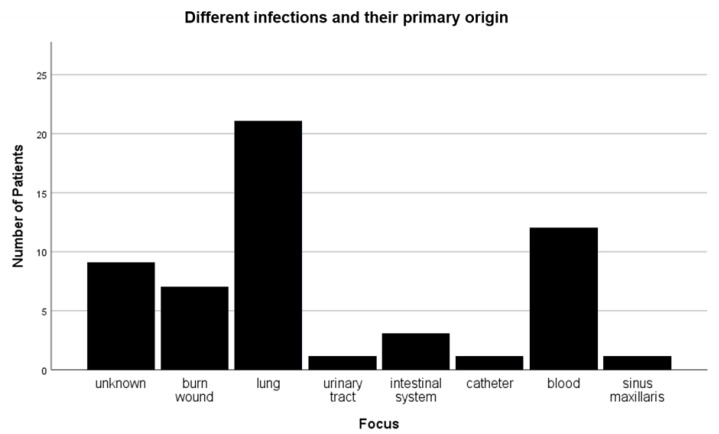
Different infections and their primary origins.

A total of 96.5% (*n* = 55) of the patients who developed an infection received surgical treatment for the burn wound. Out of these 57 patients, 9 died (15.7%). A total of 19 out of the 57 infected patients had a combination of two sources of infection. Infections most commonly originated from an initial lung infection. Other common sources of infection were bloodstream infections and wound infections (Figure 3). Interestingly, only 8 patients (38%) out of the 21 patients who were initially diagnosed with a lung infection (pneumonia) showed signs of an inhalation injury on admission. Thirteen patients with no signs of infection died during their admission, mainly due to multiple organ failure. This corresponds to 6.7% of patients with no indication of infection and 5.2% of all observed burn patients.

#### 3.2.1. Antibiotic Treatment

A rather small proportion (2.75%) of positive wound swabs showed a local infection caused by multi-resistant microbial strains, such as Acinetobacter baumanii or multi-resistant *Staphylococcus aureus*. Multi-resistant microbial strains were led by methicillin-resistant *Staphylococcus aureus* with 6 out of 255 cases.

Multi-resistant bacteria such as Acinetobacter baumanii were treated with levofloxacin or tobramycin.

A total of 112 patients (43.4%) received an intravenous antibiotic treatment, including patients who received perioperative single-shot antibiotic treatment, and 107 patients (42.5%) received some form of surgical intervention. All of the patients who underwent surgical treatment received some form of antibiotic treatment. Only five patients (1.9%) did not receive some kind of surgical intervention but still received antibiotic treatment.

On average, the first antibiotic was administered after a median time of 4 days (2–6) following admission. The median duration of antibiotic treatment was 12 days (8–21.75), with a median use of 2 (1–4) different types of antibiotics.

**Figure 4:**
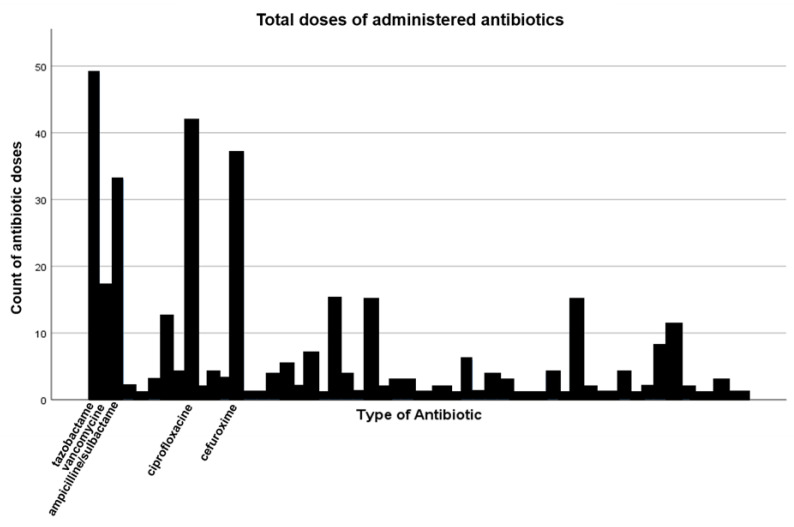
Total number of different antibiotic doses administered.

**Figure 5:**
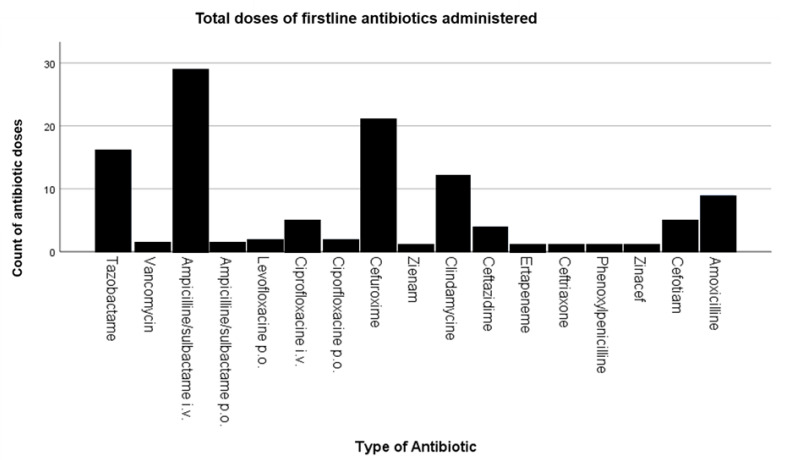
Total number of first-line antibiotic doses administered.

During the time span from 2002 until 2009, a total of 344 different antibiotics were administered. The most commonly used types of antibiotics were piperacillin/tazobactam (14.2%), followed by ciprofloxacin (12.2%), cefuroxime (10.8%), ampicillin/sulbactam (9.6%), and vancomycin (4.9%) (Figure 4). Overall, the most frequently used type of antibiotic as first-line treatment was ampicillin/sulbactam, followed by cefuroxime, piperacillin/tazobactam, clindamycin, and amoxicillin/clavulanic acid (Figure 5).

#### 3.2.2. AB Treatment Specified for Lung Infection

In 33 of the 57 patients (57.9%) with a documented focus of infection, the source was a lung infection. The median ABSI of this group was 7 (6–9.5), and the median TBSA was 25% (18–41.5). In these cases, the most frequently found microorganisms in the sputum were *Candida* spp. (22.0%), *Staphylococcus aureus* (14.0%), and *Streptococcus* (12.0%). The median duration of antibiotic administration was 18 days (13–40), with a median count of 4 different antibiotics (2–7). On average, the first antibiotic was administered after 3 days (2–5) following admission. The antibiotic primarily used to fight lung infection was piperacillin/tazobactam (14.9%), followed by ciprofloxacin (11.9%), cefuroxime (9.8%), ampicillin/sulbactam (6.3%), and vancomycin (5.6%). On average, patients with a proven lung infection spent 39 days (29.5–65) at the hospital. Three of the patients (9.1%) with infection of the lung died during their stay at the hospital.

#### 3.2.3. AB Treatment Specified for Wound Infection

In 11 out of the 57 patients (19.3%) with a documented focus of infection, the source was an infected burn wound. The median ABSI of this patient group was 6 (5–8), and the median TBSA was 19% (10–23). In these cases, the most frequently found bacterium was Pseudomonas aeruginosa, which was the case in four infected wounds. This was followed equally by *Enterococcus*, Cswabs spp., *E. coli*, coagulase-negative Staphylococcus, *Streptococcus*, Proteus, and *Staphylococcus aureus* (each found in three infected wounds). In all cases, more than a single bacterium could be detected in the wound. The median time of antibiotic administration was 13 days (7.5–35.5), with a median count of 2 (1–4) different antibiotics. The first antibiotic was administered after a median time of 5 days (1–13) after admission. The antibiotics primarily used to fight wound infection were piperacillin/tazobactam, ampicillin/sulbactam, and ciprofloxacin. The most commonly used first-line antibiotic treatment was ampicillin/sulbactam (36.4%). On average, patients with a proven wound infection spent 43 days (27–61) at the hospital. One of the patients with an infected wound died during their stay at the hospital.

### 3.3. Time Matters (2002–2005 vs. 2006–2009)

**Figure 6:**
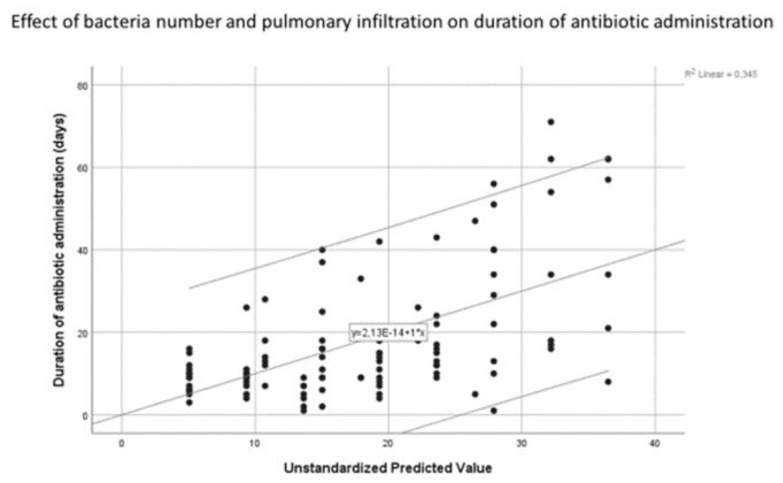
Scatter plot depicting the multivariate relationship between the duration of antibiotic administration and the independent variables bacteria number and radiological signs of pulmonary infiltration represented by the unstandardised predicted value. Line of best fit and confidence interval of 95%.

To examine whether there were any trends across time, we constructed two “year groups”, in which patients admitted between 2002 and 2005 were assigned to group 1, and patients admitted between 2006 and 2009 were assigned to group 2. When comparing the results from 2002 to 2005 to those from 2006 to 2009, it could be seen that in the time span from 2002 to 2005, a total number of 141 people (56.0%) had been treated at the burn unit. During the time frame from 2002 to 2005, a total of 160 (62.7%) positive swabs from wounds were collected, and the most commonly found bacterium in infected wounds was *Staphylococcus aureus* (11.8%, Gram-positive), followed by *Pseudomonas aeruginosa* (10.6%, Gram-negative), coagulase-negative Staphylococcus (10.0%, Gram-positive), *Enterococcus* (9.4%, Gram-positive), and *E.coli* (Gram-negative) and *Enterobacter* spp. (Gram-negative), each with 6.3% (Figure 6).

Out of these patients, 26 (18.4%) showed signs of infection and were consequently antibiotically treated. The first antibiotic treatment started after a median time of 5 days (3–8). The median duration of antibiotic treatment was still 17 days (7.5–33.25). The leading primary cause of infection was lung infection (34.6%; *n* = 9), followed by bloodstream infection (26.9%; *n* = 7) and wound infection (11.5%; *n* = 3).

The results of the Mann–Whitney U test showed that the length of hospital stay of the second group (*n* = 186) had a lower mean rank (107.64) than the first group (*n* = 66, mean rank = 179.66). This difference was statistically significant (*p* < 0.001). This indicates that group 2 generally had shorter stays compared to group 1. There also appeared to be a statistically significant difference in both TBSA (*p* < 0.001) and ABSI scores (*p* < 0.001) between the groups. The TBSA of the second group tended to be smaller (mean rank = 108.99) than that of the first group (mean rank = 173.67). The ABSI scores also appeared to be lower, on average, in the second year group (mean rank = 109.78) compared to the first year group (mean rank = 171.45).

When comparing the number and types of antibiotics administered in the two year groups, we found that in group 1, a total of 209 different antibiotics were administered to 66 patients, while in group 2, that number was 135 in 46 patients. In group 1, the most commonly used type of antibiotic was piperacillin/tazobactam (12.9%), followed by ciprofloxacin (12.4%), cefuroxime (10.5%), ampicillin/sulbactam (7.2%), and amoxicillin/clavulanic acid (5.3%). In group 2, the most commonly used type of antibiotics were piperacillin/tazobactam (16.3%), ampicillin/sulbactam (13.3%), ciprofloxacin (11.9%), cefuroxime (11.1%), and vancomycin (5.9%). When analysing first-line antibiotic treatment, regardless of infection source, ampicillin/sulbactam was, on average, the antibiotic of choice in both groups (group 1: 18.2%; group 2: 25.8%). It is interesting to note that, while amoxicillin/clavulanic acid was the fifth most commonly used type of antibiotic during the timespan from 2002 to 2005, it was not used a single time during the time span from 2006 to 2009.

In group 2, 111 people (44.0%) were treated at the hospital, and an infection was detected in 31 patients in that group (27.9%). In comparison, in group 2, a total of 95 (37.3%) positive swab samples from infected wounds were collected. The predominant bacteria were coagulase-negative Staphylococcus (15.8%, Gram-positive), followed by *Pseudomonas aeruginosa* (10.5%, Gram-negative), *E. coli* (Gram-negative) and *Enterococcus* (Gram-positive), each with 9.5%, and *Staphylococcus aureus* (8.4%, Gram-positive) (Table 3). In group 2, the first antibiotic treatment started after a median time of 4 days (2–6). The median duration of antibiotic administration during that time frame was 14.5 days (8.75–29.5). The leading primary cause of infection in these cases was infection of the lung (38.7%).

A Whitney–Mann U test showed that the number of bacteria found in the patients did not significantly differ between those admitted between 2002 and 2005 and those admitted between 2006 and 2009 (*p* = 0.395). The difference in the number of antibiotics administered to each patient was also statistically insignificant (*p* = 0.690), and the difference between the mean rank (55.07) of the second group (*n* = 46) and that of the first group (*n* = 66, mean rank = 57.50) was very small. Thus, it cannot be concluded that neither the number of bacteria found in patients nor the number of administered antibiotics differed significantly between the year groups. However, there was a significant difference between the time at which the first antibiotic was administered (*p* < 0.001). Patients in group 2 were administered the first antibiotic sooner (*n* = 186, mean rank = 101.50) than those in group 1 (*n* = 66, mean rank = 196.95). There was also a significant difference in the duration of antibiotic administration in the two groups (*p* < 0.001). The second group had a significantly shorter antibiotic administration time (*n* = 183, mean rank = 99.60) compared to the first group (*n* = 66, mean rank = 193.17).

### 3.4. Total Body Surface Area

A total of 17.9% of all patients (45 of 252) showed a TBSA greater than 30%. Of these patients, 37.8% (*n* = 17) died during their hospital stay, mostly due to multiple organ failure. Far more patients (207 of 252; 82.1%) showed a TBSA less than 30%. Of this group, 2% (*n* = 5) died—again, mostly due to multiple organ failure.

The median unit treatment time in patients with a TBSA less than 30% was 17 days (8–30.75). A source of infection was detected in 43 (20.8%) of these patients. These patients received antibiotic treatment accordingly. Treatment was begun, on average, 5 days (3–8) after admission and continued for a mean time of 13 days (7–19). Patients with a TBSA greater than 30% showed a median admission time in the burn unit of 34 days (3–63). A source of infection was detected in 14 (31.1%) of these patients. Antibiotic treatment for these patients began after a median period of 3 days (1–5) following admission and continued for 22 days (14.5–49).

The Mann–Whitney U test conducted on these two groups showed that the above-noted differences between the groups were statistically significant. The hospital stay (*p* = 0.014) was significantly longer (mean rank = 149.89) for patients with TBSA > 30% than those with TBSA < 30% (mean rank = 120.78). The duration of antibiotic treatment (*p* < 0.001) was also longer in the TBSA > 30% group (mean rank = 169.15) than in the TBSA < 30% group (mean rank = 114.75).

It should be noted that deaths in burn patients caused by multiple organ failure tend to occur within 5 days of injury [10]. As such, when focusing on deaths in the burn unit, the data show that the duration of stay in patients with TBSA over 30% increases to 56 days (range 2–151; SD 38.8), while for those with a TBSA less than 30%, it remains at 21 days (range 0–113; SD 17.6).

### 3.5. Survivors

A total of 22 patients (8.6%) died during their stay at the hospital, most of them due to multiple organ failure. The median period of time until death was 5.5 days (2–22.75). This group’s median ABSI was 12 (8–14), and their median TBSA was 52.5% (29–81.25). Out of these 22 patients, 17 had been intubated, and 9 out of the 22 patients (40.9%) had a microbiologically proven focus of infection. This corresponds to 15.8% of all patients with documented infections and 3.6% of all observed burn patients. The Mann–Whitney U test conducted on the dead-versus-alive patients showed that the differences in ABSI score and TBSA were statistically significant (both *p* < 0.001). The patients who died tended to have a higher TBSA (mean rank = 213.43) and ABSI score (mean rank = 227.68) than those who survived (TBSA mean rank = 117.60; ABSI mean rank = 116.23). The hospital stay was also significantly shorter (*p* = 0.001) amongst those who died (mean rank = 79.50) compared to those who survived (mean rank = 131.000).

### 3.6. Regression Analyses

Multiple regression was carried out using a model that predicted the duration of hospital stay by the number of bacteria, TBSA, ABSI, and the presence of inhalation injury. In this model, all patients were included—even if their swabs showed no bacterial presence. This model was able to explain 53% of the variation in the outcome variable and was a significant predictor (*p* < 0.001). The f-ratio was very high (F = 69.47), indicating that the variation among group means was higher than expected. More specifically, the model showed that for every additional bacterium found in the patient’s swabs, the stay increased by 9.55 days (B = 9.55, *p* < 0.05). The model also showed that the higher the ABSI score, the longer the duration of stay (B = 2.83, *p* < 0.05). Notably, it also showed that a higher TBSA was associated with a slightly shorter stay (B = −0.31, *p* < 0.05). Nonetheless, this can be explained by the high mortality rate amongst those with a high TBSA. Inhalation injury (B = 4.47, *p* = 0.16) was not a significant predictor of hospital stay duration.

**Figure 7:**
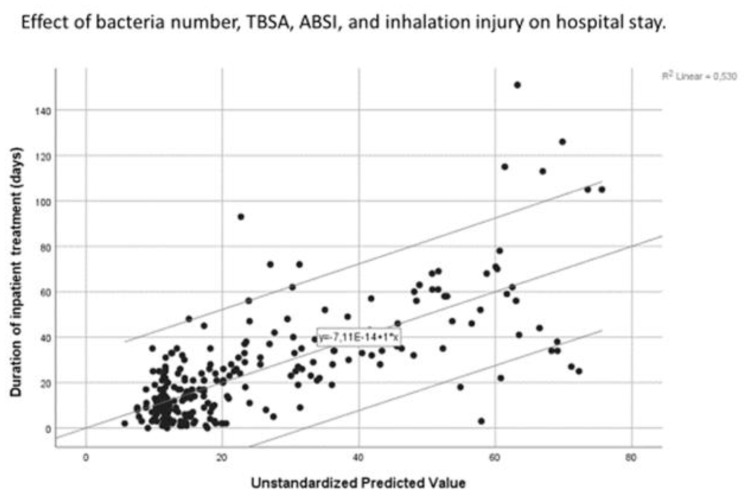
Scatter plot depicting the multivariate relationship between hospital stay duration and the independent variables bacteria number, ABSI score, TBSA, and inhalation injury, represented by the unstandardised predicted value. Line of best fit and confidence interval of 95% included.

Almost all assumptions for linearity were fulfilled in this model. Scatter plots showed that the relationship between the independent variables and the duration of hospital stay was linear (Figure 7 and Appendix I and Appendix II).

The assumption of no collinearity was met, as VIF scores were below 10, and tolerance scores were above 0.2 (between 1.17 and 3.68 and between 0.27 and 0.86, respectively). Nonetheless, it should be noted that the ABSI score was only partially dependent on TBSA, and there was therefore slight dependence between these two variables. The values of the residuals were independent, as the obtained Durbin–Watson score was close to 2 (DW-score = 1.95), and the variance of the residuals was constant, as the plot of standardised residuals versus standardised predicted values showed no indications of funnelling. Nonetheless, it should be noted that there were few observations to draw this conclusion from, and funnelling patterns may not have been clear enough to the eye. The values of the residuals do not appear to be normally distributed and thus present one violation of the assumptions of linearity. However, as there were no extreme deviations, the results are most likely still valid. Lastly, Cook’s distance values were all under 1, indicating that there were no influential outliers affecting the model.

Multiple regression was also carried out using a model that predicted the duration of antibiotic administration by the number of bacteria and the presence of pulmonary infiltrate (Figure 6).

The proposed model was able to explain 34% of the variation in the outcome variable and was a significant predictor of antibiotic administration duration (F(2, 82) = 20.83; *p* < 0.001). More specifically, the model showed that for every additional bacterium found in the patient’s swabs, the stay increased by 5.28 days (B = 5.28, *p* < 0.001). The presence of pulmonary infiltrate was also a significant predictor of antibiotic administration duration (B = 6.87, *p* = 0.049). Here, the presence of pulmonary infiltrate was associated with an increase in antibiotic administration duration of 6.87 days. All assumptions, save the assumption of normally distributed residuals, were met. Nonetheless, as only extreme deviations from normality will significantly impact the findings, the results are most likely still valid.

### 3.7. Binary Logistic Regression

Binary logistic regression analysis was carried out to determine whether the ABSI score, TBSA, and number of bacteria are good predictors of whether a patient will develop an infection or not. The three explanatory variables proved to have an explanatory power of over 33.7% of the variation in the dependent variable (Nagelkerke’s pseudo-R2 = 0.337). All explanatory variables appeared to be significant predictors of infection development at the 5% level (Chi-Square = 62.781, df = 3, and *p* < 0.001). The odds ratio for bacteria number was 1.91 (Wald = 32.579, *p* < 0.001, 95% CI 1.531–2.389), while for the ABSI score, this was 1.30 (Wald = 4.192, *p* = 0.041, 95% CI 1.011–1.681), indicating that the likelihood of infection increases by a factor of 1.91 and 1.30, respectively. For TBSA, the odds ratio was 0.962 (Wald = 4.460, *p* = 0.035, 95% CI 0.929–0.997), indicating that, as TBSA increases, an infection becomes less likely. The model was able to correctly predict 92.3% of the cases where no infection was detected and 41.1% of the cases where an infection was detected. Thus, the model has an overall correct prediction rate of 80.9%.

Binary logistic regression analysis was also carried out to determine whether the ABSI score, TBSA, and number of bacteria are good predictors of mortality in patients. All three predictors explain 58.7% of the variability in mortality in patients (Nagelkerke’s pseudo-R2 = 0.587). The results indicate that the ABSI score is a significant predictor of mortality at the 5% level (Chi-Square = 3.59, df = 1, *p* < 0.001). The odds ratio for the ABSI score was 2.73 (Wald = 15.494, *p* < 0.001, 95% CI 1.654–4.489), meaning that the likelihood of death in a patient increases by a factor of 2.73 when the ABSI score increases by one unit. Neither TBSA nor the bacteria number was a significant predictor of mortality (*p* = 0.285 and *p* = 0.771, respectively). The model was able to correctly predict 54.4% of the cases where a death occurred and 98.3% of the cases where no death occurred. Thus, the model has an overall correct prediction rate of 94.4%.

## 4. Discussion

This study relied on retrospective data to explore characteristics, trends, and differences across various variables in burn patients admitted to the RWTH Aachen University Hospital. As with most retrospective studies, the use of this type of data comes with challenges and disadvantages. Most importantly, retrospective data are not able to determine causation due to the lack of controls and confounders, making the determination of pure causation difficult.

The sample size (*n* = 252) across a time span of seven years is relatively small. Of these, only 57 developed infections, and 11 specifically developed wound infections. While this indicates a high ecological validity vis-à-vis what kind of complications a burn unit may see day-to-day and year-by-year in a real-life setting, the data are limited in their ability to draw conclusions about the optimal treatment for burn patients who develop infections. These low numbers furthermore limit the present study’s ability to make statistical inferences about the bacterial types and numbers that are related to infection. Nonetheless, the data are useful for tracking practice, such as the most used antibiotic, antibiotic administration period, etc.

The small sample size of this study may also lead to concern amongst some when it comes to the binary logistic regression carried out in the analysis of this study. Most would argue that a very large *n*—at least 500—is necessary for logistic regression. However, Leblanc and Fitzgerald argue that it is acceptable for there to be as few as 30 observations per independent variable for results from a binary logistic regression analysis to be valid [9]. Field argues that there must be at least 50 observations per predictor variable [8]. Following these guidelines, our study is expected to have revealed valid results.

Burn patients represent a unique patient cohort. Severe burns destroy the barrier between sterile tissue and the colonised external world. Microorganisms can easily spread and infiltrate necrotic tissue [11]. Avascular necrotic tissue represents a protein-rich environment that is favourable for microbial colonisation. Based on the avascularity of the burned tissue, the migration of host immune cells and the delivery of antimicrobial agents are impaired. Burn wounds present a warm and moist environment, which provides a fertile breeding ground for microorganisms. Repeated surgery and antibiotic treatment of these wounds further promote the emergence of organisms that become increasingly more difficult to fight [12]. Initial burn wounds are sterile. However, within a few days, Gram-positive strains, such as *Staphylococcus aureus*, coagulase-negative Staphylococcus, and *Streptococcus*, start to colonise the wounds from deeper structures (hair follicles and glands). In the second phase, a Gram-negative shift takes place, where Pseudomonas aeruginosa, *Escherichia coli*, and proteus are the predominant isolates [2]. If left untreated, this colonisation can lead to infection [13]. Our data showed that the number of bacteria found on the wound bed was a significant predictor of whether a patient would develop an infection. They also showed that, with each additional bacterium found in the burn wound, the stay at the hospital significantly increased by 9.5 days, and the duration of antibiotic treatment increased by 5.3 days. Especially when wound closure is delayed, multi-resistant strains can develop and lead to serious complications, longer intensive care stays, and sometimes even death [2]. Therefore, early operative treatment of wounds, consisting of the removal of the burned tissue, as well as careful selection and administration of antibiotic treatment, is crucial in treating burn patients. Wide and indiscriminate use of antibiotics is contraindicated, since it can lead to a fatal loss of infection control, as well as the proliferation of multi-resistant microbial strains [11,14], not only in burn centres but also in other intensive care units in the hospital. Such administration of antibiotics therefore does not significantly contribute to adequate wound management.

Cases in which perioperative antibiotic treatment is used during surgical intervention represent an exception to this, since therapy options such as debridement and excision can lead to bacteriaemia, where the prophylactical use of antibiotics is adequate [15]. Accordingly, patients in need of surgical intervention admitted to our burn unit all received a perioperative antibiotic treatment.

Rafla et al. pointed out that the most important factors that may trigger morbidity in and affect the mortality of burn patients are large burn wounds (>30% TBSA), significant amounts of full-thickness burns, prolonged open wounds, and delayed burn wound care [16]. This was reflected in our data, as the median TBSA of the 22 dead burn patients was 52.5%. Our dataset showed a significantly longer stay at the hospital as well as a longer duration of antibiotic administration for patients with a TBSA greater than 30% compared to the ones with a TBSA less than 30%. Relatedly, the ABSI score indicates the mortality risk in burn patients. In our study, the ABSI scores of the burn patients who died were, on average, very high at 11, corresponding to a survival rate of 20–40%. The ABSI score was also associated with infection development, a longer hospital stay, and longer antibiotic treatment duration. One interesting finding was that the likelihood of an infection decreased with increasing TBSA. While this initially may seem counterintuitive, it can be explained by the higher mortality rates in patients with a high TBSA. When putting into perspective that deaths in burn patients caused by multiple organ failure tend to occur within 5 days of injury [10], one might deduce that patients with a high TBSA tend to die before a potential infection can either be diagnosed or originate.

To successfully treat burn patients, it is crucial to quickly diagnose infections. The ABA has presented modified crucial criteria for detecting infections in burn patients. Experienced burn physicians rely on other clues as signs of infection or sepsis, such as dropping platelet counts after three days following admission, increased fluid requirements, impaired renal function, intolerance to enteral feeding, hyperglycaemia in non-diabetic patients, and altered mental status. Another sign of rising infection can be a rise in specific infection laboratory factors, such as PCT, Interleukin 6, TNF alpha (tumour necrosis factor), and others [5].

For a clear diagnosis of infection, there should be a strong distinction between wound colonisation and infection. Colonisation refers to the presence of bacteria on the wound surface at low concentrations (<105 bacteria/g tissue) with no invasive infection. A wound infection is defined as bacteria on the wound surface, as well as the wound eschar, at high concentrations (>105 bacteria/g tissue) [5]. This also applies to bacterial strains that can become multi-resistant, such as *Pseudomonas aeruginosa* [17]. Our data showed this distinction in practice, as 89 patients showed the presence of bacteria in their swabs, but only 57 developed an infection, and 11 specifically developed a wound infection. The spectrum of bacteria found to be colonising the wound bed essentially did not differ much from the commonly expected microbial strains [11], as coagulase-negative Staphylococcus, *Staphylococcus aureus*, *Pseudomonas aeruginosa*, and *Enterococcus* were the leading microorganisms in our sample size. However, the absolute distribution of bacteria in our study population did slightly differ from the literature. Future research with a greater number of wound infection occurrences than what was found in this retrospective study could valuably contribute to our knowledge with empirical evidence of which bacteria most commonly cause infections in burn patients.

Another important question refers to the dosing of antibiotics. Burn patients show unique pharmacokinetics and pharmacodynamics compared to other intensive care patients due to significant pathophysiologic changes [17]. Inefficient treatment of burn patients can be related to insufficient dosing. This has to be considered when antibiotic treatment becomes necessary. Certain studies suggest an average of 1.5 to 2 times higher dosages of antibiotics than the recommended dosage for typical patients with infections. This is recommended because burn patients eliminate drugs extremely rapidly as a consequence of augmented renal clearance and altered protein binding [2,18]. We argue that a dosage increase of the applied antibiotic should depend on the clinical situation. Drug monitoring through serum concentration measurements should be mandatory to reveal the toxicity levels of the applied drug. In our study, patients highly threatened by sepsis were treated with higher dosages, mostly 1.3 times the average recommended dosage. When comparing the number and most commonly used types of antibiotics administered in the 2002–2005 and 2006–2009 groups, it could be seen that the “tool box” of antibiotic treatments did not necessarily change much, since ampicillin/sulbactam, piperacillin/tazobactam, ciprofloxacin, and cefuroxime were among the most frequently used antibiotics throughout the entire time span. These substances, in general, are amongst the most commonly used groups of antibiotics, according to the literature [11]. Additionally, on average, the number of antibiotics used per patient, as well as the absolute count of bacteria present in burn wounds, did not differ significantly between the two year groups. However, patients being treated between 2006 and 2009 had a significantly shorter stay at the hospital, as well as shorter antibiotic treatment, which, furthermore, started earlier compared to the years 2002–2005. While these findings might be partially explainable by the, on average, milder burn cases with lower TBSA admitted to our burn unit, it could also potentially indicate that the treatment algorithm for fighting microbial colonisation in the second year group became more efficient.

Finally, beyond local invasive surgical measures and systemic antibiotic treatment, a more stratified application of local wound antiseptics may be advised. In light of the incremental rise of multidrug-resistant bacterial infections, which are also increasingly gaining relevance in burn patients [19], new strategies for local therapy have to be discussed. While only two antiseptics, namely, polyhexanide and mafenide acetate, were used in our reported patient collective, precise and well-coordinated use of other antiseptics may be favourable. Along these lines, evidence from recent studies [20] and ongoing experiments of BSK indicate the feasibility of antiseptics against multidrug-resistant bacteria, with repetitive antibacterial testing of antiseptics representing an innovative approach. Finally, clinicians are advised to direct their attention towards novel strategies, such as the implementation of phages in combination with antibiotics [21] or antiseptics (ongoing research of BSK), which again may boost antimicrobial efficacy.

## 5. Conclusions

In this paper, we study second- and third-degree adult patients admitted to the burn unit of the RWTH Aachen University Hospital. Motivated by the complexities involved in treating this unique patient cohort, our ambition was to contribute to a better understanding of the dynamics and correlations in burn treatment.

The results of the descriptive statistical analysis of this study show that there are marked differences in key parameters between burn patients who developed an infection and those who did not, those who were admitted before 2006 and those who were admitted after, those who had a TBSA greater or smaller than 30%, and patients who died or survived the burn trauma. Patients who developed infections tended to have, on average, a higher TBSA, higher ABSI scores, and longer hospital stays. Patients who were admitted to the burn unit after 2006 had significantly shorter stays at the burn unit than those admitted before. Nonetheless, TBSA and ABSI scores were lower in the patient cohort admitted after 2006, suggesting that the shorter hospital stay was due to this phenomenon. However, the duration of antibiotic treatment was significantly shorter in patients admitted after 2006, although the number of bacteria found in patients and the number of antibiotics used did not differ significantly between the two year groups. Patients exhibiting a TBSA greater than 30% had significantly longer hospital stays and antibiotic treatment periods. TBSA and ABSI scores were significantly higher in patients who died than in those who survived their burn injury.

Results of the linear regression analysis indicate that the number of bacteria, TBSA, and ABSI score are significant predictors of hospital stay duration. Here, bacteria number and the ABSI score showed a positive correlation, while TBSA showed a slight negative correlation with hospital stay duration. Bacteria number and the presence of pulmonary infiltrate proved to be significant predictors of antibiotic treatment duration, with both variables showing a positive correlation with treatment duration. This indicates that early operative treatment of wounds, consisting of the removal of the burned tissue, is crucial in treating burn patients. Lastly, the results of binary logistic regression indicate that a higher ABSI score increases the odds ratio of developing an infection and of dying. Bacteria number had no significant effect on the odds of patient death but positively influenced the odds ratio of developing an infection. TBSA was negatively associated with the risk of developing an infection and was a statistically insignificant predictor of mortality. Nonetheless, the descriptive statistics showed that all of the patients who died had a median TBSA of 52.5% and an IQR of 29–81.25.

As with all retrospective data, there is no control over covariates, and clean causal inference cannot be made on the basis of these data. Nonetheless, this study has yielded descriptive data, which provides greater insight into the correlations and causations associated with burn injury.

## Figures and Tables

**Table 1:** Infection criteria in adult burn patients (according to the American Burn Association) [5].

A minimum of 3 of the following should exist:
Temperature > 39 °C or < 36.5; Progressive tachycardia > 110 bpm; Progressive tachypnoea > 25 bpm not ventilated; Thrombocytopenia (will not apply until 3 days after admission) < 100,000/mcl; Hyperglycaemia (in the absence of pre-existing diabetes) > 200 mg/dL and/or existence of insulin resistance: (a) > 7 units of insulin/hour intravenous drip and/or (b) significant resistance to insulin (>25% increase in insulin requirements over 24 h); Inability to continue enteral feedings > 24 h: (a) abdominal extension, (b) enteral feeding intolerance, or (c) uncontrollable diarrhoea (>2500 mL/day).

**Table 2:** The presented table gives an overview of the total count of microorganisms found in positive wound swabs, as well as the respective count of each microorganism in non-infected and infected wounds.

Type of Microorganism	Count of Respective Microorganisms in All Wound Swabs	Count of Respective Microorganisms in Non-Infected Wounds	Count of Respective Microorganism in Infected Wounds
Coagulase-negative Staphylococcus	31	28	3
*Pseudomonas aeruginosa*	27	23	4
*Staphylococcus aureus*	27	24	3
*Enterococcus* spp.	24	21	3
*Escherichia coli*	22	19	3
*Enterobacter* spp.	17	16	1
*Klebsiella pneumoniae*	17	15	2
*Streptococcus*	16	13	3
*Candida* spp.	15	12	3
*Bacillus* spp.	9	8	1
Gram-negative rods	9	8	1
*Proteus mirabilis*	8	5	3
*Serratia marascensens*	6	4	2
Methicillin-resistant *Staphylococcus aureus*	6	4	2
*Klebsiella oxytoca*	6	5	1
*Staphylococcus epidermidis*	4	3	1
*Acinobacter baumanii*	3	3	0
*Aspergillus* spp.	1	1	0
*Herpes simplex virus*	1	1	0
*Klebsiella ornithinolytica*	1	1	0
*Morganella morganii*	1	1	0
*Citrobacter farmii*	1	1	0
*Sphingomonas paucimobilis*	1	1	0
*Citrobacter freundii*	1	1	0
*Aeromonas hidrophila*	1	1	0
**Total**	255	219	36

**Table 3:** The presented table gives an overview of the kinds of microorganisms found in positive wound swabs and their absolute count in positive wound swabs in the timespans of 2002 to 2005 and 2006 to 2009.

Type of Microorganism Found in Positive Wound Swabs	Count of Microorganisms Found in Positive Wound Swabs between 2002 and 2005	Count of Microorganisms Found in Positive Wound Swabs between 2006 and 2009
*Staphylococcus aureus*	19	8
*Pseudomonas aeruginosa*	17	10
Coagulase-negative Staphylococcus	16	15
*Enterococcus* spp.	15	9
*Escherichia coli*	13	9
*Enterobacter* spp.	13	4
*Streptococcus*	11	5
*Klebsiella pneumoniae*	10	7
*Candida* spp.	9	6
Gram-negative rods	7	2
*Bacillus* spp.	7	2
*Proteus mirabilis*	5	3
Methicillin-resistant *Staphylococcus aureus*	4	2
*Klebsiella oxytoca*	4	2
*Serratia marascensens*	3	3
*Acinobacter baumanii*	1	2
*Aspergillus* spp.	1	0
*Staphylococcus epidermidis*	1	3
*Sphingomonas paucimobilis*	1	0
*Citrobacter freundii*	1	0
*Aeromonas hidrophila*	1	0
*Morganella morganii*	1	0
*Klebsiella ornithinolytica*	0	1
*Herpes simplex virus*	0	1
*Citrobacter farmii*	0	1
**Total**	160	95

## Appendix A. SPSS Output Multivariate Regression Analysis—Hospital Stay Duration Model

**Table A1:** Model Summary.

Model	R	R-Squared	Adjusted R-Squared	Std. Error of the Estimate
1	0.728 (a)	0.530	0.523	16.419

(a) Predictors: (Constant), bacteria number, inhalation injury, TBSA, ABSI score.

**Table A2:** ANOVA (a).

Model		Sum of Squares	df	Mean Square	F	Sig
1	Regression	74,902.867	4	18,725.717	69.465	0.000 (b)
	Residual	66,314.209	246	296.570		
	Total	141,217.076	250			

(a) Dependent Variable: Duration of stay. (b) Predictors: (Constant), bacteria number, inhalation injury, TBSA, ABSI score.

**Table A3:** Coefficients (a).

		Non-Standardised Coefficients		Standardised Coefficients			95% Confidence Interval for B	
Model		B	Std. Error	Beta	T	Sig	Lower Bound	Upper Bound
1	(Constant)	−4.002	7.659		−0.523	0.602	−19.088	11.084
	TBSA	−0.310	0.103	−0.252	−3.012	0.003	−0.512	−0.107
	ABSI	2.834	0.791	0.326	3.582	0.000	1.275	4.392
	Inhalation injury	2.834	3.181	0.070	1.406	0.161	−1.794	10.736
	Bacteria number	4.471	0.710	0.670	13.454	0.000	8.150	10.946

(a) Dependent Variable: Duration of hospital stay.

## Appendix B. SPSS Output Multivariate Regression Analysis—Antibiotic Treatment Duration

**Table A4:** Model Summary.

Model	R	R-Squared	Adjusted R-Squared	Std. Error of the Estimate
1	0.580 (a)	0.337	0.321	13.786

(a) Predictors: (Constant), bacteria number, presence of pulmonary infiltrate.

**Table A5:** ANOVA (a).

Model		Sum of Squares	df	Mean Square	F	Sig
1	Regression	7916.823	2	3958.412	20.828	0.000 (b)
	Residual	15,584.165	82	190.051		
	Total	23,500.988	84			

(a) Dependent Variable: Duration of antibiotic treatment. (b) Predictors: (Constant), bacteria number, presence of pumonary infiltrate.

**Table A6:** Coefficients (a).

		Non-Standardised Coefficients		Standardised Coefficients			95% Confidence Interval for B	
Model		B	Std. Error	Beta	T	Sig	Lower Bound	Upper Bound
1	(Constant)	0.498	3.489		−0.143	0.887	−6.444	7.439
	Pulmonary infiltrate	6.868	3.433	0.186	2.001	0.049	0.039	13.696
	Bacteria number	5.276	0.971	0.505	5.434	0.000	3.344	7.207

(a) Dependent Variable: Duration of antibiotic administration.
